# Supplementary material for: Identification of novel vertebral development factors through UK Biobank candidate gene search and body imaging analysis
Source: Commun Biol. 2025 Nov 19;8:1614. doi: 10.1038/s42003-025-09021-8 (PMC12630705; doi:10.1038/s42003-025-09021-8)
Supplement: Supplementary file 1 — Supplementary materials [file 42003_2025_9021_MOESM1_ESM.pdf]

Supplementary Materials for

Identification of novel vertebral development factors through UK Biobank candidate gene search and body imaging analysis

Zhuopin Sun and Jiru Han et al.

*Corresponding author. Email: bahlo@wehi.edu.au

This PDF file includes:

Supplementary Figures 1 to 7
Supplementary Tables 1 to 4

Supplementary Figure 1.

Associations between vertebral anomalies and physical measures including the standing height, sitting height, seated height, waist, and hip circumferences, FEV and FVC. Error bars indicate 95% confidence intervals of the effect sizes, and adjusted p-values are shown. Red lines highlight significant positive associations with FDR adjusted p-values < 0.05. Blue lines highlight significant negative associations. ***: p < 0.001; **: p < 0.01; *: p < 0.05.

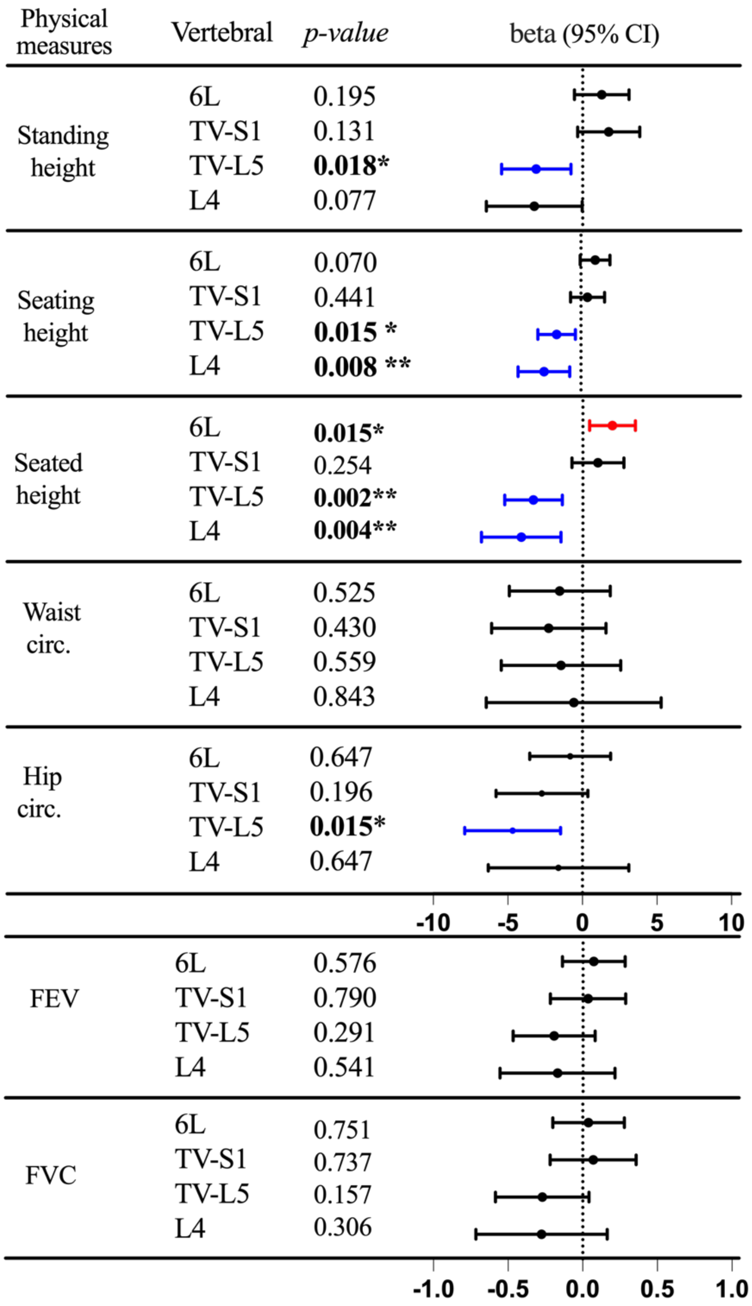

Supplementary Figure 2.

Associations between rib anomalies and the experience of pain lasting 3+ months (including headaches, neck/shoulder pain, and back pain). Odds ratio (OR) are calculated based on logistic regression models with sex and age as covariates. Odds ratios are presented on a log scale, with error bars indicating 95% confidence intervals. FDR adjusted one-sided *p*-values are displayed. Abbreviations: L1R: extra ribs on L1; 12R-hypo: hypoplastic 12th ribs; 11R: 11 rib pairs.

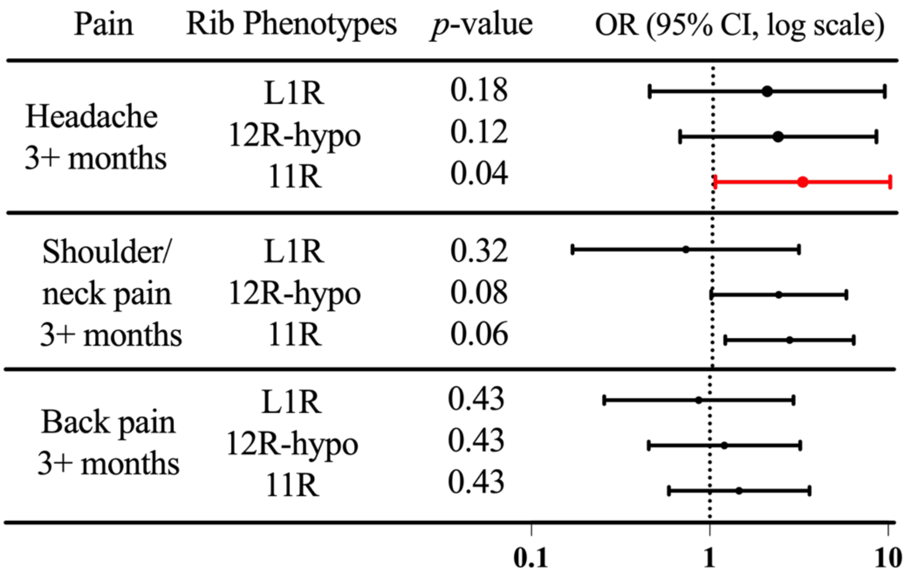

Supplementary Figure 3.

Associations between variant status and the experience of pain lasting 3+ months (including headaches, neck/shoulder pain, and back pain). Error bars indicating 95% confidence intervals (CI) of odds ratio (OR). No test reaches a statistically significant threshold (FDR adjusted *p*-values < 0.05). Abbreviations: Heterozygous = Het; Hemizygous = Hemi. Only *GPC3* has Hemi as it is the only gene on the X chromosome.

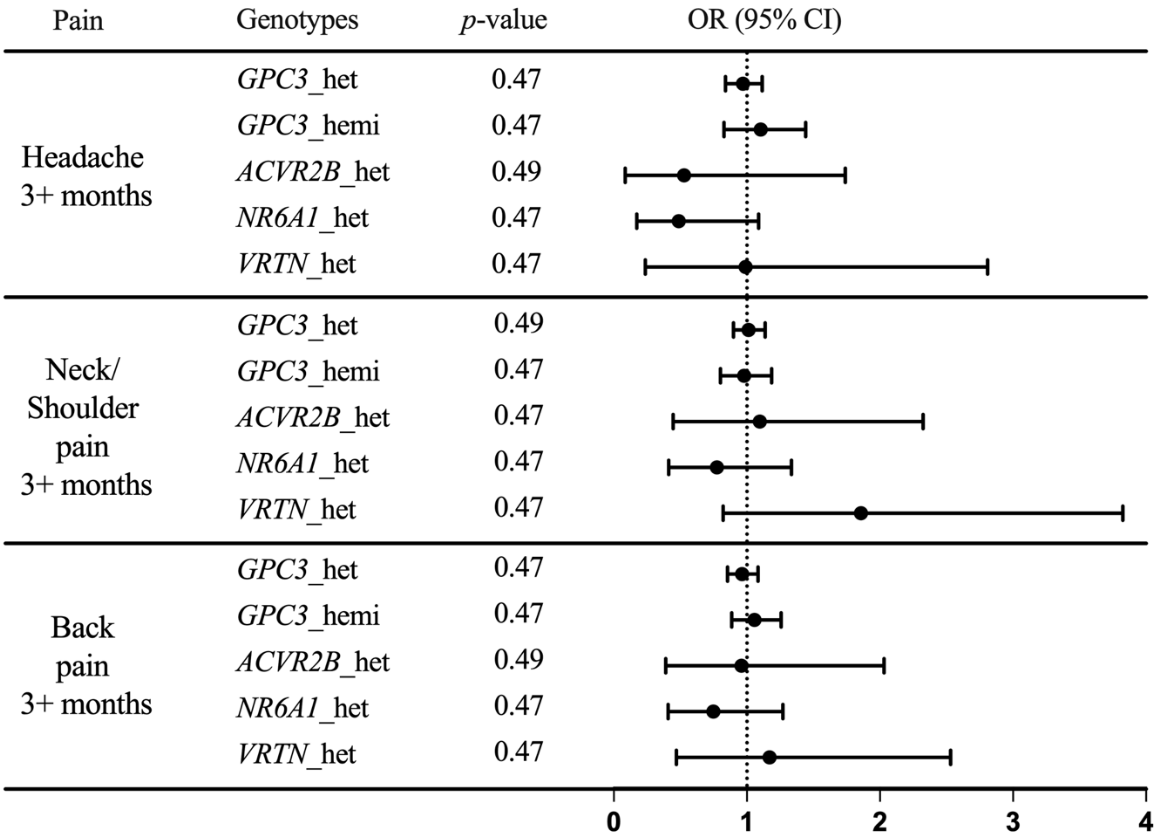

Supplementary Figure 4.

Association between chronic pain outcomes (headache, neck/shoulder pain, and back pain for 3+ months) and predictor variables, including genetic variant carriers in green (grouped *GPC3*, *VRTN*, *ACVR2B*, and *NR6A1* variants) and vertebral anomalies in orange (grouped six lumbar vertebrae, transitional S1, complete sacralization, and four lumbar vertebrae). One-sided tests were performed, and *p*-values were adjusted using the false discovery rate (FDR) method. Error bars indicate 95% confidence intervals (CI) of the odds ratios (OR), and adjusted p-values are shown. A significant association (*FDR-adjusted p < 0.05) is observed between vertebral anomalies and chronic headache.

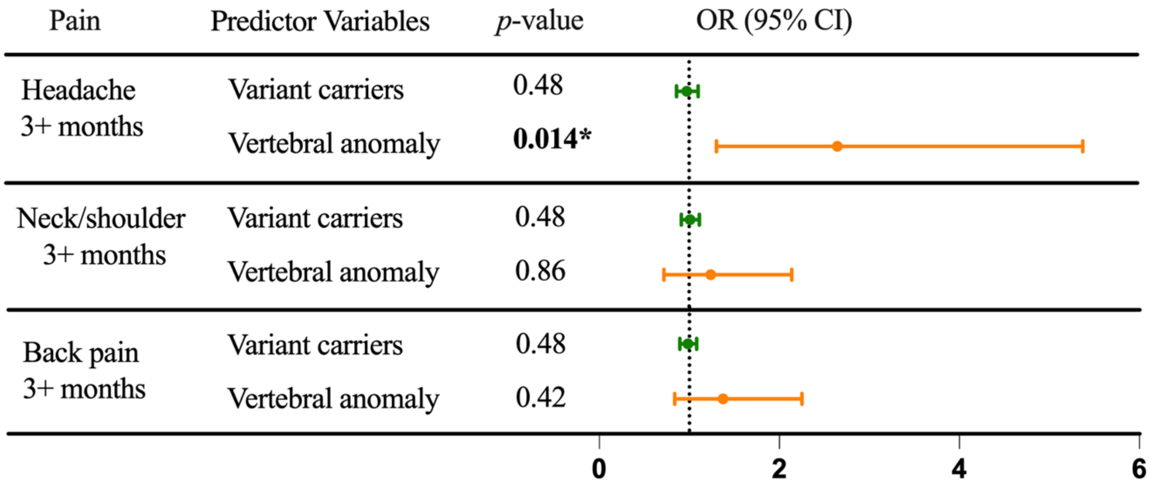

Supplementary Figure 5.

A schematic representation of the candidate gene and variant selection process. A gene panel was first selected through known clinical or phenotypic associations. (A) Genes collected from the Human Phenotype Ontology (HPO) database. (B) Genes collected from the Mouse Genome Informatics (MGI) phenotype databases, along with additional gene *NR6A1* identified in our previous mouse studies.

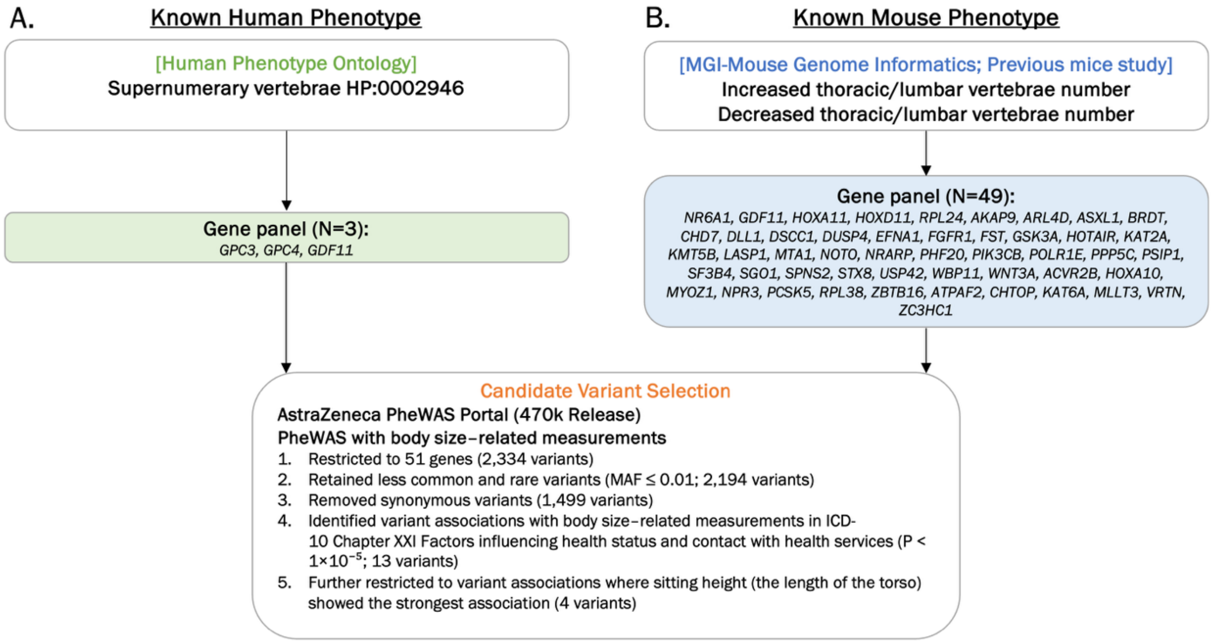

Supplementary Figure 6.

Flowchart for vertebral and rib phenotyping. (A) When C2 is visible on the body MRI, vertebrae are counted sequentially from C2 down to the last presacral segment. (B) When C2 is not visible on the MRI, alignment between DXA and MR scans is achieved through dense correspondence matching. Intervertebral discs are labelled on the MRI and overlaid onto the whole-body DXA scan. T1 is identified according to the location of the first rib pairs on the DXA images. Vertebral and rib phenotypes are then annotated accordingly. Reproduced by kind permission of UK Biobank ©.

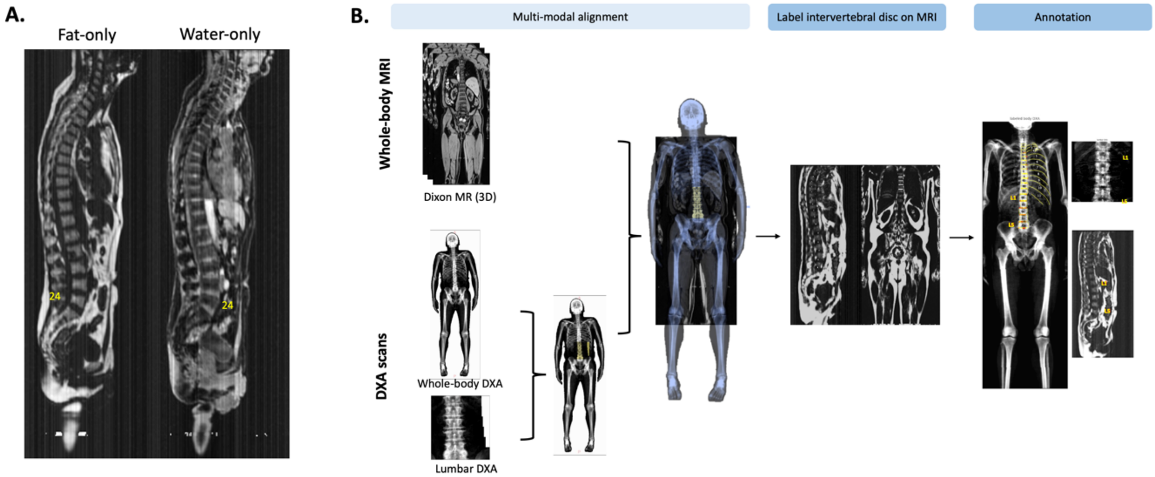

Supplementary Figure 7.

Example Dixon MR and DXA (total body and lumbar) images illustrating the vertebral and rib phenotypes assessed in this study. (A) Five vertebral phenotypes, presented from the most to the least number of vertebral segments: 6 lumbar vertebrae, lumbarised S1, normal (5 lumbar vertebrae), complete sacralisation, and 4 lumbar vertebrae. Additional or fewer vertebral segments are indicated by red and cyan arrows, respectively. (B) Rib phenotypes, examined based on high-resolution lumbar DXA images, include the presence of extra L1 ribs, 12 rib pairs, hypoplasia of the 12th ribs, and aplasia of the 12th ribs. Additional or fewer ribs compared to the normal 12 rib pairs are indicated by magenta and green arrows, respectively. Reproduced by kind permission of UK Biobank ©.

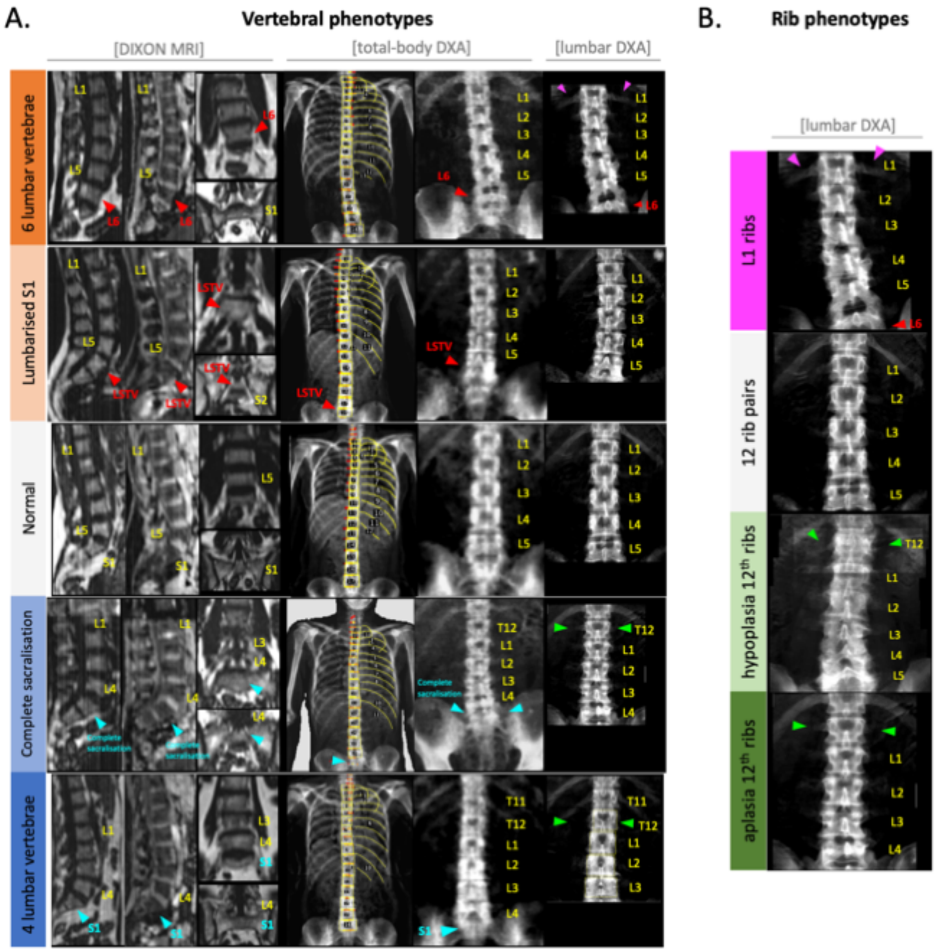

Supplementary Table 1.

Mann-Whitney U test results comparing the distribution of vertebral and rib phenotypes between males and females across different genotypes. The table presents the phenotype type, genotypes, Mann-Whitney U statistic, and p-values for each test. No comparison reaches a statistically significant threshold.

**Phenotype**	**Genotype**	**Mann-Whitney U Statistic**	***p-value***
Vertebral	Non-carriers	31839.50	0.52
Vertebral	*ACVR2B* het	40.00	0.28
Vertebral	*NR6A1* het	27.50	0.09
Vertebral	*VRTN* het	3.50	0.81
Ribs	Non-carriers	30781.50	0.54
Ribs	*ACVR2B* het	30.00	0.28
Ribs	*NR6A1* het	12.50	0.44
Ribs	*VRTN* het	3.50	0.81

Supplementary Table 2.
Association between vertebral phenotypes and health-related outcomes. Logistic regression was used to estimate the association between vertebral anomaly categories (reference: normal anatomy) and disease outcomes, with sex as a covariate. Adjusted p-values are reported after two levels of false discovery rate (FDR) correction: (1) within each disease for four vertebral phenotypes, and (2) within each ICD-10 subcategory (i.e. G90-G99: Other disorders of the nervous system). Significant p-values after the second FDR correction are highlighted in bold. Sample sizes for each vertebral phenotype category were as follows: normal anatomy (n = 805), 6 lumbar vertebrae (n = 49), transitional S1 (n = 38), complete sacralisation (n = 35), and 4 lumbar vertebrae (n = 15). *: adjusted P-value<0.05 **: adjusted P-value<0.01.

	Six lumbar	Transitional S1	Complete sacralisation	Four lumbar
Nervous system disorders	G93 Other disorders of brain	G50 Disorders of trigeminal nerve		G24 Dystonia
Eye and adnexa disorders	**H02 Other disorders of eyelid***	**H52 Disorders of refraction and accommodation***		
Ear and mastoid process disorders				H61 Other disorders of external ear
Circulatory system disorders				I25 Chronic ischaemic heart disease<br>I87 Other disorders of veins
Respiratory system disorders			J01 Acute sinusitis<br>J04 Acute laryngitis and tracheitis	
Digestive system disorders			K64 Haemorrhoids and perianal venous thrombosis	K06 Other disorders of gingiva and edentulous alveolar ridge<br>K22 Other diseases of oesophagus<br>**K44 Diaphragmatic hernia****<br>K59 Other functional intestinal disorders<br>**K92 Other diseases of digestive system***
Musculoskeletal system and connective tissue disorders	M13 Other arthritis		**M47 Spondylosis***	M47 Spondylosis
Genitourinary system disorders		N13 Obstructive and reflux uropathy	N34 Urethritis and urethral syndrome	N34 Urethritis and urethral syndrome

-

Supplementary Table 3.

Summary of associations between health-related outcomes and the four variant carrier status. Logistic regression was used to estimate the association between variant carrier status and disease outcomes. Sex and ancestry are included as covariates. Adjusted p-values are reported after false discovery rate (FDR) correction for the four variants within each ICD-10 subcategory (i.e. G90-G99: Other disorders of the nervous system). *: adjusted p-value<0.05; **: adjusted p-value<0.01.

**Category**	***GPC3*** **Carriers**	***NR6A1*** **Carriers**	***VRTN*** **Carriers**	***ACVR2B*** **Carriers**
Digestive system disorders			K13: other diseases of lip and oral mucosa**	
Eye and adnexa disorders				H25: senile cataract*
Genitourinary system disorders		N76: other inflammation of vagina and vulva*		
Musculoskeletal system and connective tissue disorders				M46: other inflammatory spondylopathies**
Respiratory system disorders	J40: bronchitis, not specified as acute or chronic*		J43: emphysema*<br>J45: asthma*	

Supplementary Table 4.

Variants associated with body size–related measurements. Thirteen variants met the significance threshold ($P < 1\times10^{-5}$) in AZPheWAS analyses and were associated with body size–related measurements (ICD-10 Chapter XXI: Factors influencing health status and contact with health services).

Gene	Variant	Phenotype	Model	Consequence type	MAF	Effect size	p-value
ACVR2B	3-38483237-C-T	20015#Sitting height	dominant	missense_variant	0.00015	-0.344	2.83E-07
			genotypic			-0.344	2.83E-07
		50#Standing height	dominant			-0.290	3.64E-06
			genotypic			-0.290	3.64E-06
NPR3	5-32780776-C-T	50#Standing height	dominant	missense_variant	5.68E-05	0.512	5.67E-07
			genotypic			0.512	5.67E-07
	5-32782990-G-A		dominant		0.0037	0.064	5.13E-07
			genotypic			0.063	5.48E-07
	5-32784801-G-A	20015#Sitting height	dominant	missense_variant&splice_region_variant	0.0023	0.096	1.36E-08
			genotypic			0.096	1.35E-08
		50#Standing height	dominant			0.147	1.88E-20
			genotypic			0.146	2.21E-20
		51#Seated height	dominant			0.094	9.07E-08
			genotypic			0.094	8.30E-08
	5-32786307-C-T	50#Standing height	dominant	missense_variant	0.0013	0.114	8.22E-08
			genotypic			0.114	8.22E-08
AKAP9	7-92065227-A-G	50#Standing height	dominant	splice_region_variant	0.0046	0.056	1.45E-06
			genotypic			0.057	1.10E-06
NR6A1	9-124524718-T-C	20015#Sitting height	dominant	splice_region_variant	0.0001	-0.319	5.97E-06
			genotypic			-0.319	5.97E-06
		51#Seated height	dominant			-0.329	6.24E-06
			genotypic			-0.329	6.24E-06
POLR1E	9-37486618-A-G	23112#Leg fat mass (right)	dominant	missense_variant	3.03E-05	0.698	9.71E-06
			genotypic			0.698	9.71E-06
PCSK5	9-76193240-CG-C	50#Standing height	dominant	frameshift_variant	2.39E-05	-0.694	9.54E-06
			genotypic			-0.694	9.54E-06
VRTN	14-74357184-G-A	20015#Sitting height	dominant	missense_variant	4.42E-05	-0.562	4.98E-06
			genotypic			-0.562	4.98E-06
KAT2A	17-42117515-T-C	50#Standing height	dominant	missense_variant	0.00053	0.151	5.45E-06
			genotypic			0.151	5.45E-06
PPP5C	19-46347098-T-C	21002#Weight	dominant	start_lost	0.00064	-0.174	4.12E-06
			genotypic			-0.172	4.37E-06
			dominant			-0.172	5.05E-06

			genotypic			-0.171	5.35E-06
		23099#Body fat percentage	dominant			-0.147	6.42E-06
			genotypic			-0.146	6.75E-06
		23100#Whole body fat mass	dominant			-0.197	2.32E-06
			genotypic			-0.196	2.46E-06
		23111#Leg fat percentage (right)	dominant			-0.124	3.67E-06
			genotypic			-0.122	4.15E-06
		23112#Leg fat mass (right)	dominant			-0.159	3.49E-06
			genotypic			-0.157	3.74E-06
		23115#Leg fat percentage (left)	dominant			-0.128	1.24E-06
			genotypic			-0.126	1.41E-06
		23116#Leg fat mass (left)	dominant			-0.163	1.54E-06
			genotypic			-0.161	1.68E-06
		23127#Trunk fat percentage	dominant			-0.178	5.38E-06
			genotypic			-0.176	5.67E-06
		23128#Trunk fat mass	dominant			-0.203	2.30E-06
			genotypic			-0.202	2.43E-06
GPC3	X-133692376-C-T	20015#Sitting height	dominant	missense_variant	0.0044	0.072	4.34E-07
			genotypic			0.057	3.02E-08
			recessive			0.122	2.34E-06
		50#Standing height	genotypic			0.043	9.59E-06
		51#Seated height	genotypic			0.052	1.04E-06
			recessive			0.135	4.15E-07
