# Supplementary material for: Identification of novel vertebral development factors through UK Biobank candidate gene search and body imaging analysis
Source: Commun Biol. 2025 Nov 19;8:1614. doi: 10.1038/s42003-025-09021-8 (PMC12630705; doi:10.1038/s42003-025-09021-8)
Supplement: Supplementary file 4 — Reporting summary [file 42003_2025_9021_MOESM4_ESM.pdf]

nature portfolio

Corresponding author(s): Melanie Bahlo

Last updated by author(s): Sep 1, 2025

Reporting Summary

Nature Portfolio wishes to improve the reproducibility of the work that we publish. This form provides structure for consistency and transparency in reporting. For further information on Nature Portfolio policies, see our Editorial Policies and the Editorial Policy Checklist.

Statistics

For all statistical analyses, confirm that the following items are present in the figure legend, table legend, main text, or Methods section.

n/a	Confirmed	
☐	☒	The exact sample size (*n*) for each experimental group/condition, given as a discrete number and unit of measurement
☐	☒	A statement on whether measurements were taken from distinct samples or whether the same sample was measured repeatedly
☐	☒	The statistical test(s) used AND whether they are one- or two-sided<br>*Only common tests should be described solely by name; describe more complex techniques in the Methods section.*
☐	☒	A description of all covariates tested
☐	☒	A description of any assumptions or corrections, such as tests of normality and adjustment for multiple comparisons
☐	☒	A full description of the statistical parameters including central tendency (e.g. means) or other basic estimates (e.g. regression coefficient) AND variation (e.g. standard deviation) or associated estimates of uncertainty (e.g. confidence intervals)
☒	☐	For null hypothesis testing, the test statistic (e.g. *F*, *t*, *r*) with confidence intervals, effect sizes, degrees of freedom and *P* value noted<br>*Give P values as exact values whenever suitable.*
☒	☐	For Bayesian analysis, information on the choice of priors and Markov chain Monte Carlo settings
☒	☐	For hierarchical and complex designs, identification of the appropriate level for tests and full reporting of outcomes
☐	☒	Estimates of effect sizes (e.g. Cohen's *d*, Pearson's *r*), indicating how they were calculated

Our web collection on statistics for biologists contains articles on many of the points above.

Software and code

Policy information about availability of computer code

Data collection: No software was used for data collection.

Data analysis: The image pre-processing and alignment packages are publicly available at: https://github.com/rwindsor1/UKBiobankDXAMRIPreprocessing
https://github.com/rwindsor1/biobank-self-supervised-alignment
R (v4.1.2)

For manuscripts utilizing custom algorithms or software that are central to the research but not yet described in published literature, software must be made available to editors and reviewers. We strongly encourage code deposition in a community repository (e.g. GitHub). See the Nature Portfolio guidelines for submitting code & software for further information.

Data

Policy information about availability of data

All manuscripts must include a data availability statement. This statement should provide the following information, where applicable:

- Accession codes, unique identifiers, or web links for publicly available datasets
- A description of any restrictions on data availability
- For clinical datasets or third party data, please ensure that the statement adheres to our policy

All WES, physical measurements, reported pain data, and imaging data described in this paper are publicly available to registered researchers through the UKB data-

access protocol. Additional information about registration for access to the data are available at: https://www.ukbiobank.ac.uk/enable-your-research/apply-for-access. Further information about the WES data is available at https://www.ukbiobank.ac.uk/media/najcnoaz/access_064-uk-biobank-exome-release-faq_v11-1_final-002.pdf. Detailed information about UK biobank imaging is available at https://biobank.ndph.ox.ac.uk/ukb/ukb/docs/body_mri_explan.pdf for body MRI and https://biobank.ctsu.ox.ac.uk/crystal/crystal/docs/DXA_explan_doc.pdf for DXA imaging.

Research involving human participants, their data, or biological material

Policy information about studies with human participants or human data. See also policy information about sex, gender (identity/presentation), and sexual orientation and race, ethnicity and racism.

Reporting on sex and gender	Sex was determined using genetic data. We obtained sex information from UK Biobank field Data-Field 22001. Sex was included as a covariate in all analyses.<br>For the primary analyses in this paper, 486 (51.6%) individuals were female, 456 (48.4%) were male.
Reporting on race, ethnicity, or other socially relevant groupings	Our analyses used ancestry information provided by the Pan-UKBB resource (https://pan.ukbb.broadinstitute.org)
Population characteristics	Among participants with WES data in this study, the mean age at recruitment was 56.5 years (SD = 8.1), with 54.1% female. Within the UK Biobank imaging sub-cohort (N = 76,277), the mean age at imaging was 65.5 years (SD = 7.8), and 51.6% were female. Age and sex characteristics of candidate variant carriers with body imaging included are described in details in Table 3. A control group of 500 age- and sex-matched non-carriers (50% female) was selected to represent the broader UKB imaging population.
Recruitment	The UK Biobank is a prospective cohort study of over 500,000 individuals from across the United Kingdom, aged 40–69 years at recruitment. A wide range of phenotypic information and biological samples were collected at baseline, together with linkage to electronic health records. A subset of participants underwent body imaging, comprising dual-energy X-ray absorptiometry (DXA) and whole-body MRI.
Ethics oversight	Genetic and imaging data from the UK Biobank were accessed under application number 36610. The UK Biobank has ethical approval from the UK National Health Service National Research Ethics Service. This research was additionally approved by the Walter and Eliza Hall Institute of Medical Research Human Research Ethics Committee (HREC 17/09LR)

Note that full information on the approval of the study protocol must also be provided in the manuscript.

Field-specific reporting

Please select the one below that is the best fit for your research. If you are not sure, read the appropriate sections before making your selection.

☒ Life sciences ☐ Behavioural & social sciences ☐ Ecological, evolutionary & environmental sciences

For a reference copy of the document with all sections, see nature.com/documents/nr-reporting-summary-flat.pdf

Life sciences study design

All studies must disclose on these points even when the disclosure is negative.

Sample size	The sample size was not pre-determined; instead, analyses used available UK Biobank dataset with both whole-exome sequencing (WES) and body imaging (DXA and MRI).
Data exclusions	Analyses were restricted to carriers of candidate rare variants with genetic and body imaging data, passing quality control. A control group of 500 age-matched non-carriers was selected. Individuals who withdrew or were related were excluded.
Replication	No formal replication in independent cohorts was undertaken due to the lack of suitable datasets with genetic and vertebral imaging data. Analyses were limited to the UK Biobank cohort, with internal control groups (non-carriers) selected to reflect baseline population characteristics.
Randomization	This was an observational study, and no randomization was undertaken.
Blinding	No blinding was undertaken for this study. Group allocation (variant carrier vs. non-carrier) was based on genetic data.

Reporting for specific materials, systems and methods

We require information from authors about some types of materials, experimental systems and methods used in many studies. Here, indicate whether each material, system or method listed is relevant to your study. If you are not sure if a list item applies to your research, read the appropriate section before selecting a response.

Materials & experimental systems

n/a	Involved in the study
☒	☐ Antibodies
☒	☐ Eukaryotic cell lines
☒	☐ Palaeontology and archaeology
☒	☐ Animals and other organisms
☒	☐ Clinical data
☒	☐ Dual use research of concern
☒	☐ Plants

Methods

n/a	Involved in the study
☒	☐ ChIP-seq
☒	☐ Flow cytometry
☒	☐ MRI-based neuroimaging

Plants

Seed stocks	*Report on the source of all seed stocks or other plant material used. If applicable, state the seed stock centre and catalogue number. If plant specimens were collected from the field, describe the collection location, date and sampling procedures.*
Novel plant genotypes	*Describe the methods by which all novel plant genotypes were produced. This includes those generated by transgenic approaches, gene editing, chemical/radiation-based mutagenesis and hybridization. For transgenic lines, describe the transformation method, the number of independent lines analyzed and the generation upon which experiments were performed. For gene-edited lines, describe the editor used, the endogenous sequence targeted for editing, the targeting guide RNA sequence (if applicable) and how the editor was applied.*
Authentication	*Describe any authentication procedures for each seed stock used or novel genotype generated. Describe any experiments used to assess the effect of a mutation and, where applicable, how potential secondary effects (e.g. second site T-DNA insertions, mosiacism, off-target gene editing) were examined.*
